# Interleukin-6 induces impairment in human subcutaneous adipogenesis in obesity-associated insulin resistance

**DOI:** 10.1007/s00125-016-4031-3

**Published:** 2016-06-24

**Authors:** Shamma Almuraikhy, Wael Kafienah, Moataz Bashah, Ilhame Diboun, Morana Jaganjac, Fatima Al-Khelaifi, Houari Abdesselem, Nayef A. Mazloum, Mohammed Alsayrafi, Vidya Mohamed-Ali, Mohamed A. Elrayess

**Affiliations:** 1 Anti-Doping Lab Qatar, Sports City Road, P.O. Box 27775, Doha, Qatar; 2 School of Cellular and Molecular Medicine, University of Bristol, Bristol, UK; 3 Bariatric and Metabolic Surgery, Hamad Medical Corporation, Doha, Qatar; 4 Department of Physiology and Biophysics, Weill Cornell Medicine Qatar, Doha, Qatar; 5 Microbiology and Immunology, Weill Cornell Medicine Qatar, Doha, Qatar

**Keywords:** Adipogenesis, Insulin resistance, Insulin sensitivity, Interleukin-6, Obesity, Subcutaneous fat, Type 2 diabetes mellitus

## Abstract

**Aims/hypothesis:**

A subset of obese individuals remains insulin sensitive by mechanisms as yet unclear. The hypothesis that maintenance of normal subcutaneous (SC) adipogenesis accounts, at least partially, for this protective phenotype and whether it can be abrogated by chronic exposure to IL-6 was investigated.

**Methods:**

Adipose tissue biopsies were collected from insulin-sensitive (IS) and insulin-resistant (IR) individuals undergoing weight-reduction surgery. Adipocyte size, pre-adipocyte proportion of stromal vascular fraction (SVF)-derived cells, adipogenic capacity and gene expression profiles of isolated pre-adipocytes were determined, along with local in vitro IL-6 secretion. Adipogenic capacity was further assessed in response to exogenous IL-6 application.

**Results:**

Despite being equally obese, IR individuals had significantly lower plasma leptin and adiponectin levels and higher IL-6 levels compared with age-matched IS counterparts. Elevated systemic IL-6 in IR individuals was associated with hyperplasia of adipose tissue-derived SVF cells, despite higher frequency of hypertrophied adipocytes. SC pre-adipocytes from these tissues exhibited lower adipogenic capacity accompanied by downregulation of *PPARγ* (also known as *PPARG*) and *CEBPα* (also known as *CEBPA*) and upregulation of *GATA3* expression. Impaired adipogenesis in IR individuals was further associated with increased adipose secretion of IL-6. Treatment of IS-derived SC pre-adipocytes with IL-6 reduced their adipogenic capacity to levels of the IR group.

**Conclusions/interpretation:**

Obesity-associated insulin resistance is marked by impaired SC adipogenesis, mediated, at least in a subset of individuals, by elevated local levels of IL-6. Understanding the molecular mechanisms underlying reduced adipogenic capacity in IR individuals could help target appropriate therapeutic strategies aimed at those at greatest risk of insulin resistance and type 2 diabetes mellitus.

## Introduction

Obesity has become a global healthcare priority due to the comorbidities that accompany excessive adiposity. However, a number of studies have identified subsets of obese individuals who have fewer cardiovascular risk factors than predicted by their BMI and who are often referred to as insulin-sensitive (IS) or metabolically healthy obese [1]. These individuals maintain insulin sensitivity [1, 2] and exhibit a less pro-atherogenic lipoprotein profile and less inflammation and hypertension, despite excessive body fat [3, 4]. Currently, key protective factors accounting for this healthy phenotype are unclear.

In obesity, exacerbated fat accumulation within adipose tissues is initially facilitated through local adipocyte hypertrophy [5, 6], followed by recruitment of pre-adipocytes, especially within the larger subcutaneous (SC) depots [7, 8]. Further energy intake leads to visceral fat accumulation, often accompanied by ectopic deposition in tissues such as liver, skeletal muscle and heart tissues, causing increased risk of insulin resistance and type 2 diabetes mellitus [9]. The ability of the SC depot to efficiently act as a buffer, protecting against accumulation of fat in visceral tissues, is key to preventing development of associated pathologies [10].

Pre-adipocytes (CD73^+^, CD105^+^, CD166^+^, CD11b^−^, CD14^−^, CD31^−^) comprise 15–50% of cells within adipose tissue and have the ability to differentiate into mature adipocytes (adipogenesis) in response to nutrient excess [11–13]. In obesity, the number of pre-adipocytes is inversely related to adipocyte size, predisposing to insulin resistance [6, 14, 15] independent of sex and body fat level [16]. Accordingly, tissues from insulin resistant individuals (IR) are marked with fewer, but larger, adipocytes, perhaps reflecting impaired adipogenesis [17, 18].

Many factors are thought to mediate adipocyte dysfunction in obesity, including tissue oxidative stress and inflammation [19, 20]. Lipid-engorged adipocytes secrete greater levels of cytokines, such as IL-6, IL-β, IL-8 and monocyte chemoattractant protein-1 [5, 21, 22], which can inhibit adipocyte differentiation [10]. Adipose IL-6 secretion is markedly increased in vivo in obesity, along with systemic elevation, especially in IR individuals [23–25]. Chronic treatment of rodent pre-adipocytes, 3T3-F442A and 3T3-L1, with IL-6 increased their autocrine IL-6 secretion and promoted insulin resistance consistent with downregulation of *Glut4* in adipocytes and other pro-adipogenic nuclear factors such as peroxisome proliferator-activated receptor γ (*Pparγ*, also known as PPARG) and CCAAT/enhancer-binding protein α (*Cebpα*; also known as *CEBPA*) [26].

Comparing the adipogenic capacity of SC pre-adipocytes between IS and IR individuals and examining the impact of local IL-6 release on this process in primary cultures would provide valuable insight into the potentially protective mechanism associated with the IS group. The aims of the study were to test two hypotheses: (1) the adipogenic capacity of SC pre-adipocyte is abrogated in all IR individuals compared with IS individuals; (2) this lesion is accompanied by greater release of IL-6 by pre-adipocytes that directly inhibits adipogenesis.

## Methods

### Materials

IL-6, leptin and adiponectin ELISAs and human recombinant IL-6 were from R&D systems (Abingdon, UK). Anti-human antibodies (anti-CD31-FITC, anti-CD166-PerCP-efluor) were from eBioscience (Hatfield, UK), anti-CD105-APC, anti-CD45-Alexa fluor 700 and anti-CD11b-Brilliant Violet 421 were from Biolegend (Cambridge, UK) and anti-CD73-Brilliant Violet 605 was from BD Bioscience (Oxford, UK). Insulin ELISA was from Mercodia Diagnostics (Uppsala, Sweden). DAPI, LipidTOX Green Neutral Lipid, Inflammatory Cytokine Human Magnetic 5-Plex and Trizol were from Life Technologies (Warrington, UK). RT2 Profiler human adipogenesis PCR arrays and cDNA synthesis kits were from SABiosciences-Qiagen (Hilden, Germany). Human Oxidative Stress Magnetic Bead Panel was from Millipore (Watford, UK). Human white SC-pre-adipocytes from a lean individual (C-12730) were from PromoCell (Heidelberg, Germany). Other chemicals and reagents were from Sigma (Munich, Germany).

### Participants

Obese and morbidly obese individuals (37 women/20 men) of Arab origin, mainly from Qatar, were recruited from patients undergoing weight reduction surgery at Hamad Medical Corporation (Doha, Qatar). Inclusion criteria were severe obesity (BMI ≥35 kg m^-2^) with co-morbidities including hypertension, chronic obstructive pulmonary disease, type 2 diabetes mellitus, sleep apnoea and arthritis, and morbid obesity (BMI ≥40 kg m^-2^) with no co-morbidities. Informed written consent was obtained from all subjects prior to surgery. Protocols were approved by the Institutional Review Boards of Hamad Medical Corporation (SCH-JOINT-111) and Anti-Doping Lab Qatar (SCH-ADL-070) and were carried out in accordance with the Declaration of Helsinki as revised in 2008. Blood and SC adipose tissues biopsies (1–5 g) were collected during surgery and immediately transported to the laboratory for processing. Systemic lipids, fasting plasma glucose (FPG) and liver-function enzymes were measured using a routine chemistry analyser (Cobas; Roche Diagnostics, Mannheim, Germany). IL-6, leptin, adiponectin and insulin were determined using commercially available ELISAs. Insulin resistance was calculated by HOMA-IR [27] with 2.4 as a cut-off point (30th percentile). Accordingly, individuals were dichotomised into IS (HOMA-IR <2.4, *n* = 16) and IR (HOMA-IR >2.4, *n* = 41) groups, the latter including 14 individuals with type 2 diabetes mellitus (seven treated with metformin, six treated with metformin/insulin and one treated by dietary modification).

### Isolation of stromal vascular fraction cells from human SC adipose tissue

Stromal vascular fraction (SVF) was obtained by collagenase digestion as previously described [28] and cell number was quantified per gram of tissue. The SVF pellet was re-suspended in stromal medium (DMEM-F12 containing 10% [vol./vol.] FBS, 100 units/ml penicillin and 0.1 mg/ml streptomycin) and maintained at 37°C with 5% CO_2_ until confluence, then passaged at 2 × 10^4^ cells/cm^2^ when necessary.

### Analysis of surface epitopes by FACS

Expression of various cell surface markers was performed as described previously [29]. Briefly, fixed SVF-derived pre-adipocytes were incubated with one of the following mouse anti-human antibodies: CD31-FITC (1:200), NG2-PE (1:200), CD166-PerCP-efluor (1:40), CD105-APC (1:20), CD45-Alexa fluor 700 (1:100), CD11b-Brilliant Violet 421(1:40) or CD73-Brilliant Violet 605 (1:20) in 0.5% (wt/vol.) BSA blocking solution. Non-specific mouse IgG was substituted for the primary antibody as isotype control. Analysis was performed on FACSCantoII flow cytometer (BD Bioscience) and analysed with FlowJo Software v7.6.5 (Three Star, Ashland, OR, USA). Positive expression was defined as a level of fluorescence greater than 95% of that of the corresponding isotype-matched control antibody.

### Differentiation assays

SVF-derived cells (passages 1–3) were grown in stromal medium overnight then incubated in differentiation medium (DMEM-F12 containing 3% (vol./vol.) FBS, 33 μmol/l biotin, 17 μmol/l d-pantothenate, 1 μmol/l dexamethasone, 250 μmol/l methylisobutylxanthine, 0.1 μmol/l human insulin and 5 μmol/l of PPARγ agonist, rosiglitazone) for 7 days, followed by 12 days in maintenance medium containing the same components as the differentiation medium, except for methylisobutylxanthine and rosiglitazone [30].

### Determination of adipocyte size

Adipose tissue sections were made from paraffin blocks and stained with haematoxylin/eosin. Areas of adipocytes were measured in ten random fields per sample, without prior knowledge of the experimental groups, using application suite-v4 of a light microscope (Leica Microsystems, Buffalo Grove, IL, USA).

### Viability and differentiation capacity in IS vs IR adipocytes

Total number of nuclei (DAPI positive) and differentiated adipocytes (Lipidtox positive), as well as average sizes of differentiated adipocytes, were scored in 20 fields per well by ArrayScan XTI (Life Technologies, Grand island, NY, USA) using the automated spot detection module. Cell viability was assessed by comparing the number of cells (stained nuclei) at 1 day post seeding with that scored following completion of differentiation. Differentiation capacity was assessed by calculating the number of Lipidtox-positive cells per total number of stained nuclei and presented as a percentage (adipogenic capacity). This was also validated in pre-adipocytes obtained from a lean individual who showed greater than 80% adipogenic capacity (data not shown). For investigating IL-6-mediated inhibition of differentiation, cells were grown as above, with or without 20 ng/ml IL-6 for the entire differentiation/maintenance periods.

### Antioxidant profiling of IS v IR differentiated adipocytes

The activity of the reactive oxygen species (ROS) scavenging enzymes catalase, superoxide dismutase 1 (SOD1), SOD2, peroxiredoxin 2 (PRX2) and thioredoxin (TRX1) was measured in equal concentrations of lysates from differentiated pre-adipocytes of randomly selected IS and IR individuals using Human Oxidative Stress Magnetic Bead Panel (H0XSTMAG-18 K) according to manufacturer’s instructions and assessed by Luminex Flexmap 3D using xPONENT 4.2 software (Austin, TX, USA).

### Local cytokine secretion

The supernatant media of SC pre-adipocyte cultures from IS and IR participants were collected following completion of differentiation. Accumulated levels of secreted IL-6, IL-1β, TNFα and IL-8 in the last 4 days before staining were measured using Inflammatory Cytokine Human Magnetic 5-Plex (Life Technologies) according to manufacturer’s instructions and assessed by Luminex Flexmap 3D using xPONENT 4.2 software.

### Expression studies

RNA was extracted from SC pre-adipocyte cultures before induction of differentiation, and on completion, using Trizol following manufacturer’s instructions. RT^2^ Profiler human adipogenesis PCR arrays were used to simultaneously examine the mRNA levels of 89 genes (including five housekeeping genes) in 384-well plates according to manufacturer’s protocol. Genes included *PPARγ*, *CEBPα HSL* (encoding hormone-sensitive lipase), *LEP* (encoding leptin), *SREBF1* (encoding regulatory element-binding transcription factor 1), *GLUT4* and *GATA3* (encoding trans-acting T cell-specific transcription factor). Data were normalised with internal housekeeping genes and ΔΔC_t_ was calculated using ΔC_t_ from proliferating IS SC pre-adipocytes as the control group.

### Statistical analysis

Comparisons were performed with the *t* test, Wilcoxon–Mann–Whitney test, one-way ANOVA or stepwise linear regression model as appropriate using IBM SPSS statistics 21 (Armonk, NY, USA). Significance was defined as *p* ≤ 0.05. Non-parametric tests were used for comparing ordinal or non-normal variables. Power calculations indicated that sample size (*N* = 57) had 100% power to detect a minimal difference of 30% in mean SC differentiation capacity of IS vs IR and type 2 diabetes mellitus with 34% deviation from mean value (σ) at a level of α = 0.01.

## Results

### General characteristics of study population

Fifty-seven obese and morbidly obese (BMI 43.3 ± 6.6 kg/m^2^), relatively young (35.9 ± 10.2 years), individuals of Arab origin undergoing weight reduction surgery were included. Overall, participants were hyperinsulinaemic (median [interquartile range] 79.9 [52.1–124.3] pmol/l), but were then dichotomised into IS and IR groups based on their HOMA-IR. Accordingly, 28% were insulin sensitive and 72% were insulin resistant, including 25% with known type 2 diabetes mellitus (Table 1). Compared with age- and BMI-matched IS participants, all IR (non-diabetic IR + type 2 diabetes mellitus) individuals had higher plasma levels of total cholesterol, triacylglycerols, FPG and insulin. They also exhibited higher plasma levels of liver-function enzymes alanine transaminase (ALT) and aspartate aminotransferase (AST). Basic patient characteristics are summarised in Table 1.

**Table 1:** General characteristics of study participants

Variable	Non-diabetic individuals			All IR individuals		
	IS (*n* = 16 [6 M, 10 F])	IR (*n* = 27 [(10 M, 17 F])	IS vs IR *p* value	T2DM (*n* = 14 [(4 M, 10 F])	IR + T2DM (*n* = 41, [14 M, 27 F])	IS vs IR + T2D *p* value
Age (years)	36.6 (11.8)	33.9 (8.1)	0.3	41.2 (10.8)	36.4 (9.7)	0.96
BMI (kg/m^2^)	42.9 (5.0)	44.3 (7.1)	0.57	43.5 (7.1)	44.0 (7.0)	0.55
SBP (mmHg)	127.1 (18.3)	126.9 (23.2)	0.69	128.2 (10.2)	127.3 (19.6)	0.98
DBP (mmHg)	71.29 (9.5)	72.78 (14.6)	0.98	73.86 (8.9)	73.2 (12.8)	0.62
MAP (mmHg)	89.90 (9.0)	90.81 (14.6)	0.85	92.4 (9.0)	91.4 (12.9)	0.70
Cholesterol (mmol/l)	4.48 (0.9)	5.0 (0.9)	0.53	5.10 (0.7)	5.0 (0.8)	0.04
LDL-cholesterol (mmol/l)	2.75 (0.6)	3.21 (1.0)	0.94	2.95 (0.8)	3.1 (0.9)	0.16
Triacylglycerol (mmol/l)	1.0 (0.8–1.4)	1.3 (0.9–1.9)	0.50	1.6 (1.3–2.8)	1.4 (1.0–2.1)	0.02
HDL-cholesterol (mmol/l)	1.25 (0.29)	1.20 (0.22)	0.15	1.24 (0.29)	1.2 (0.24)	0.67
FPG (mmol/l)	4.9 (0.5)	5.8 (0.9)	0.11	9.58 (5.81)	7.3 (3.8)	<0.01
Insulin (pmol/l)	50.7 (34.0–59.03)	99.3 (79.2–145.2)	<0.01	99.3 (71.5–138.2)	99.3 (78.5–141.7)	<0.01
HOMA-IR	1.5 (1.1–1.9)	3.9 (3.0–6.6)	<0.01	6.0 (3.4–9.0)	4.3 (3.0–7.2)	<0.01
Albumin (g/l)	41.5 (36.3–44.0)	42.0 (38.5–45.5)	0.40	42.0 (39.8–44.3)	42.0 (39.0–44.0)	0.34
ALP (U/l)	67.0 (57.0–102.0)	73.0 (58.5–82.3)	0.48	83.5 (60.5–117.3)	74.0 (59.8–85.8)	0.85
ALT (U/l)	17.0 (11.0–21.0)	23.0 (19.0–27.0)	0.03	29.5 (13.8–39.8)	24.0 (17.0–45.0)	<0.01
AST (U/l)	18.0 (16.0–19.0)	23.5 (15.0–29.3)	0.02	21.5 (14.0–32.5)	23.0 (15.0–30.8)	0.05
Bilirubin (μmol/l)	8.0 (6.5–13.0)	8.0 (5.8–10.3)	0.54	5.0 (4.0–7.3)	6.5 (5.0–9.0)	0.17

Data are presented as mean (SD) or median (interquartile range)

Obese and morbidly obese male and female patients were recruited and dichotomised into three groups (IS, IR and T2DM)

Components of the metabolic syndrome were measured in IS, IR, T2DM and all IR (IR + T2DM) individuals including BMI, SBP, DBP, MAP, LDL-cholesterol, HDL-cholesterol, FPG, HOMA-IR, ALP, ALT and AST

Differences between (IS vs IR) and (IS vs all IR) were tested by the independent-sample *t* test or Mann–Whitney *U* test

ALP, alkaline phosphatase; DBP, diastolic blood pressure; F, female sex; FPG, fasting plasma glucose; M, male sex; MAP, mean arterial blood pressure; SBP, systolic blood pressure; T2DM, type 2 diabetes mellitus

### Insulin resistance-associated adipokine profiling, SVF hyperplasia and adipocyte hypertrophy

**Fig. 1:**
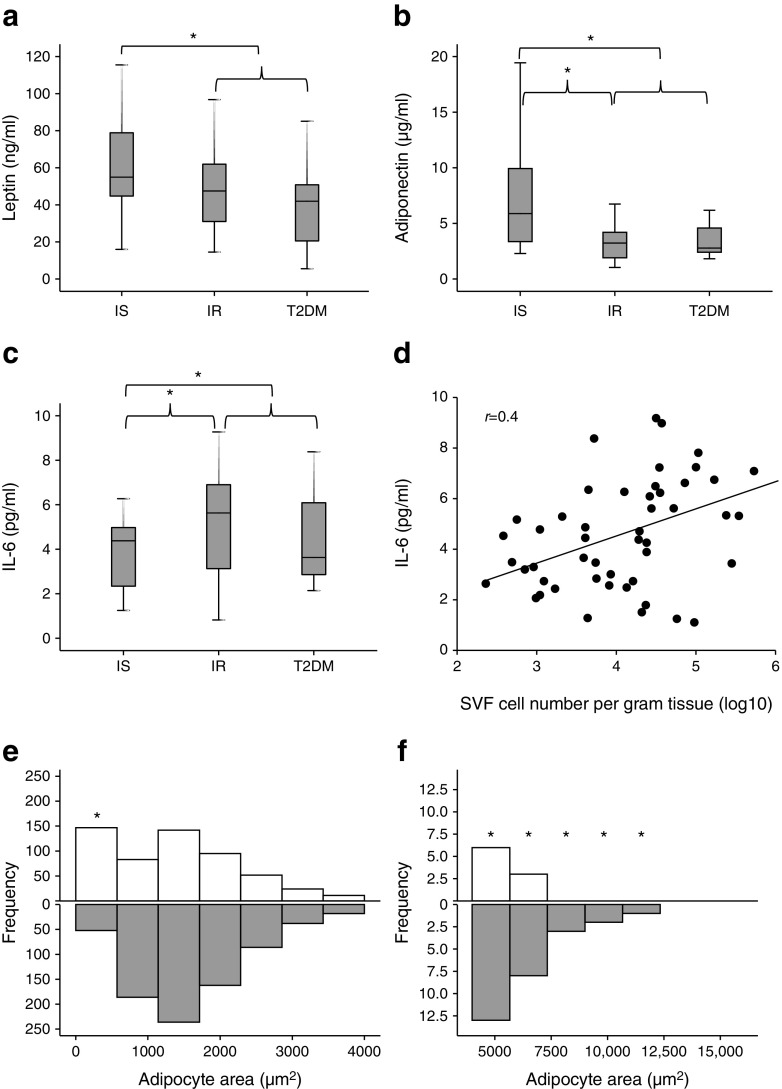
Insulin resistance-associated adipokine secretion, SVF cell numbers and adipocyte size. (**a**–**c**) Systemic levels of leptin (**a**), adiponectin (**b**) and IL-6 (**c**) were assessed in IS and IR individuals and in participants with type 2 diabetes mellitus (T2DM). (**d**) Plasma IL-6 concentrations correlated with SVF cell number per gram of adipose tissue. (**e**, **f**) Adipocyte sizes within adipose tissues from IS (white bars) and IR (grey bars) individuals were compared; frequencies of small adipocytes and large adipocytes are shown in (**e**) and (**f**), respectively. Data are presented as median ± interquartile range (*N* = 57, as shown in Table 1) in (**a**–**c**) or frequency histogram (*n* = 10: IS, *n* = 5; IR, *n* = 5) in (**e**, **f**). Differences between groups were tested by ANOVA followed by independent-sample *t* test to compare IS vs IR and IS vs all IR; *p < 0.05 (**a**–**c**). Relationship between circulating IL-6 and SVF cell number was performed with Pearson’s correlation (**d**). A rank-based two-sample Mann–Whitney *U* test was used to compare frequencies of detected sizes in IS vs IR (**e**, **f**). *p < 0.05

Compared with IS individuals, all IR (IR + type 2 diabetes mellitus) individuals exhibited a significant reduction in plasma leptin (Fig. 1a) and adiponectin (Fig. 1b), but had increased IL-6 levels (Fig. 1c). IL-6 levels were positively correlated with SVF cell number per gram tissue (*r* = 0.4, *p* = 0.04, Fig. 1d), suggesting SVF hyperplasia with increased systemic IL-6 levels. Non-diabetic IR individuals had increased number of SVF cells by 62% (*p* = 0.05), compared with IS individuals (data not shown), perhaps reflecting a greater inflammatory component or different cellular characteristics of IR SVF cells.

Assessment of adipocyte size in randomly selected adipose tissues derived from IS and IR individuals revealed a heterogeneity of sizes in both groups with greater frequencies of small adipocytes (<500 μm^2^) in IS tissues and large adipocytes (>5000 μm^2^) in IR tissues (Fig. 1e, f) (*p* < 0.01). Whereas the former suggests more plasticity of IS adipocytes, the latter confirms adipocyte hypertrophy of IR.

### Cell surface markers of the pre-adipocyte phenotype in SVF-derived cells

**Fig. 2:**
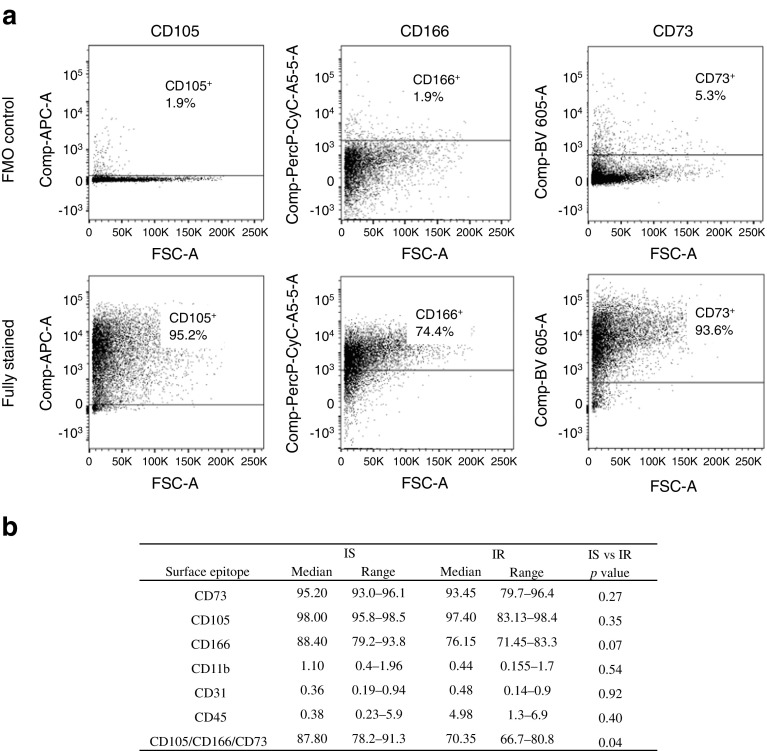
Cell surface determinants of pre-adipocyte phenotype in SVF. Results are based on FACS quantification of triple-positive population (CD105^+^, CD166^+^, CD73^+^) of SVF-derived pre-adipocytes. (**a**) Representative images indicate IR sample stained with anti-CD105, anti-CD166, anti-CD73 and corresponding fluorescence minus one (FMO) controls. (**b**) Differences between IS and IR samples are summarised in the table. Data are presented as median and interquartile range (*n* = 10: IS, *n* = 5; IR, *n* = 5) and differences between groups were tested using the Mann–Whitney *U* test

Expression of cell surface markers of the pre-adipocyte phenotype (CD105^+^, CD166^+^, CD73^+^, CD31^−^, CD11b^−^, CD45^−^) were investigated in SVF of randomly selected IS and IR adipose tissue samples. Data indicated a significantly greater proportion of pre-adipocytes in IS compared with IR samples (88% [78–91%] vs 70% [67–81%]; median [range]; *p* = 0.04) (Fig. 2a, b). This 18% reduction in pre-adipocyte pool may reflect a reduced plasticity of IR-derived cells, which may affect their adipogenic capacity.

### Insulin resistance-associated impairment of pre-adipocyte adipogenic capacity

**Fig. 3:**
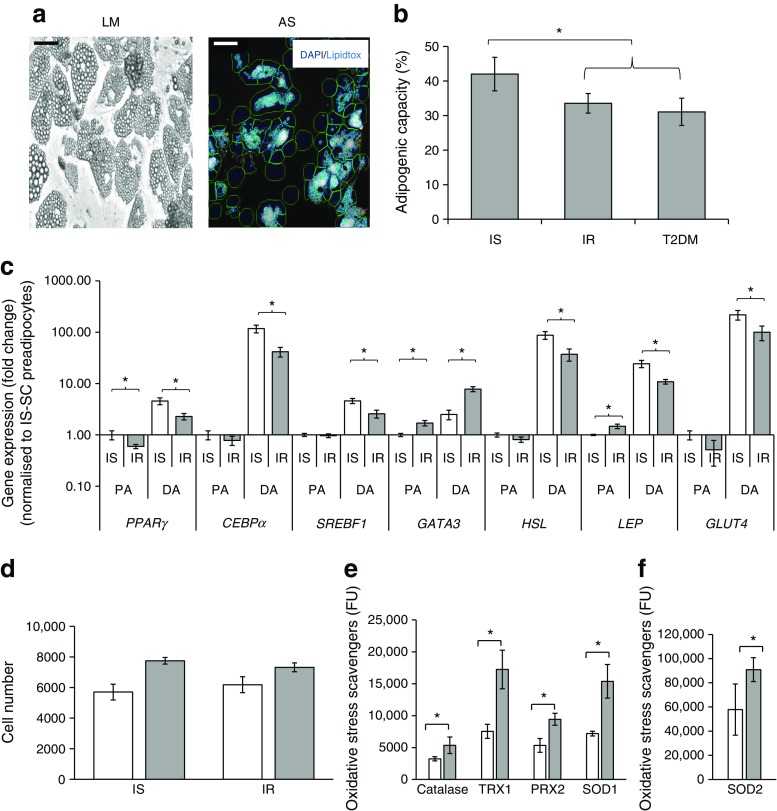
Adipogenic capacity, gene expression profile and viability of differentiated pre-adipocytes derived from IS and IR individuals. Differentiation of pre-adipocytes from IS and IR individuals and those with type 2 diabetes mellitus (T2DM) was quantified by scoring the percentage of Lipidtox-positive cells to total number of cells (adipogenic capacity). (**a**) Representative light-microscopy (LM) images of differentiated adipocytes (scale bar, 200 μm) and Arrayscan (AS; scale bar, 100 μm); the selected fluorescently labelled lipid droplets are shown. (**b**) Adipogenic capacity of the pre-adipocytes. (**c**–**f**) The expression profile of genes encoding adipogenic nuclear factors and markers of mature adipocytes was determined in pre-adipocytes (PA) and differentiated adipocytes (DA) (**c**), together with cell viability (white bar, seeding density; grey bar, density at day 19) (**d**) and activity of oxidative stress scavenging enzymes (fluorescence units, FU) in IS (white bar) and IR (grey bar)-derived cells (**e, f**). Data are presented as mean ± SEM; *N* = 57 as in Table 1 in (**b**) and *n* = 15 (IS, *n* = 5; IR, *n* = 10) in (**c**–**f**). Differences between IS and IR were tested by independent-sample *t* test; *p < 0.05

The adipogenic capacity of SVF-derived pre-adipocytes was assessed in all participants. The pre-adipocytes derived from all IR (non-diabetic IR + type 2 diabetes mellitus) individuals exhibited a reduced SC adipogenic capacity (by 27%, *p* = 0.05) compared with those from IS individuals (Fig. 3a, b). However, differentiated adipocyte size (1133 ± 237 vs 1085 ± 93.9 pixels, *p* = 0.6) and average droplet size (64.5 ± 55.2 vs 55.1 ± 32.8 pixels, *p* = 0.7) were similar in IS vs all IR individuals (data not shown). Impairment of adipogenic capacity of IR-derived pre-adipocytes was associated with downregulation of pro-adipogenic nuclear factors *PPARγ*, *CEBPα* and *SREBF1* and upregulation of anti-adipogenic nuclear factor *GATA3* pre-and post-induction of differentiation (Fig. 3c). Impairment was also marked by downregulation of mature adipocyte markers *HSL*, *LEP* and *GLUT4* (Fig. 3c). This is the first report of impairment in pre-adipocyte adipogenic potential in insulin resistance, independent of obesity, in humans. Differences in adipogenic capacity between insulin sensitivity and insulin resistance did not reflect reduced cell viability in insulin resistance (Fig. 3d) despite increased markers of oxidative stress (Fig. 3e, f).

### Role of IL-6 in impairment of pre-adipocyte adipogenesis

To identify mediators of SC differentiation, beyond hyperinsulinaemia, regression models with variables in Table 1 were carried out after exclusion of insulin and HOMA-IR. The models revealed systemic IL-6 to be the main predictor in all groups (IS + IR + type 2 diabetes mellitus) (β = −0.3, *p* = 0.03), including the IR (β = −0.4, *p* = 0.05) and non-diabetic groups (IS + IR) (β = −0.4, *p* = 0.01), explaining 10%, 20% and 20% of SC differentiation, respectively (data not shown). This suggests a role for IL-6 as a marker or mediator of impaired adipogenesis.

**Fig. 4:**
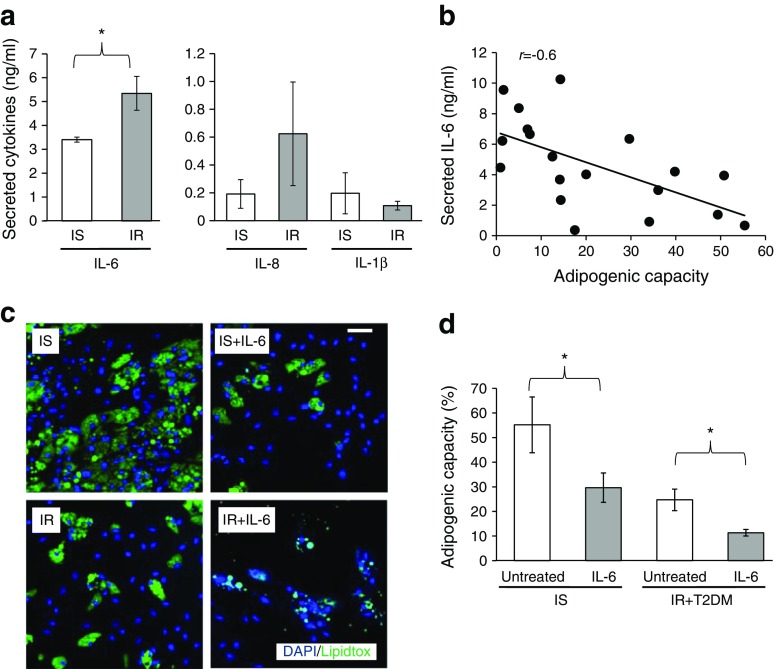
Role of IL-6 in the impairment of pre-adipocyte adipogenic capacity. (**a**) Secreted levels of IL-6, IL-8 and IL-1β were compared between IS- and IR-derived differentiated adipocytes. (**b**) Correlation between in vitro secreted IL-6 and adipocyte differentiation capacity. (**c**, **d**) Effect of treatment with 20 ng/ml IL-6 throughout differentiation on adipogenic capacity (representative images; scale bar, 100 μm (**c**) were quantified in IS and IR + type 2 diabetes mellitus (IR + T2DM) groups (**d**). Data are presented as mean ± SEM (*n* = 21 [IS, *n* = 7; IR, *n* = 14). Differences in paired groups were tested by paired-samples *t* test (**a**, **d**) and the relationship between secreted IL-6 and adipogenic capacity was examined with Pearson’s correlation (**b**); *p < 0.05

To investigate the role of IL-6 in adipogenesis, local IL-6 secretion was determined in cultured pre-adipocytes from IS and IR individuals. Compared with IS-derived cultures, all IR (IR + type 2 diabetes mellitus) SC-derived cultures secreted higher levels of IL-6 (3.4 ± 3.1 vs 6.8 ± 1.8 ng/ml, respectively). Secreted levels of IL-1β, IL-8 and TNFα were also compared between IS- and IR-derived differentiated adipocytes but neither IL-1β nor IL-8 differed significantly between the two groups (Fig. 4a). TNFα concentrations were below the limit of detectability.

Secreted IL-6 and SC adipogenic capacity were inversely correlated (*r* = −0.6, *p* < 0.01) (Fig. 4b), suggesting that elevated secretion of IL-6 may play a role in SC differentiation. SC-secreted IL-6 also exhibited a negative correlation with *CEBPα* expression (*r* = −0.6, *p* = 0.03) and a positive correlation with *GATA3* expression (*r* = 0.6, *p* = 0.06) (data not shown).

To confirm whether IL-6 inhibits SC differentiation in IS- and IR-derived pre-adipocytes, the adipogenic capacity of these cells was assessed in the presence of exogenous IL-6 (20 ng/ml). Indeed, treatment of IS-derived cultures with IL-6 reduced SC adipogenic capacity by 46% (*p* = 0.01) compared with the capacity of untreated cells, despite there being no change in cell number (*p* = 0.9), to a level similar to that of the IR group. Differentiation of the IR group was further reduced by 54% (*p* < 0.01) by IL-6 treatment, with no significant effect on cell number (*p* = 0.4) (Fig. 4c, d). Thus, the association between impaired adipogenesis and both systemic and secreted IL-6 levels in the IR group suggested a role for IL-6 in abrogating SC adipogenesis, which was confirmed by IL-6 intervention studies.

## Discussion

Emerging data points to loss of adipogenic capacity of SC pre-adipocytes and alteration in the phenotype of this depot, with increased hyperplasia of the stromal cells and hypertrophy of adipocytes being important in the development of insulin resistance and predisposition to type 2 diabetes mellitus [21, 31, 32]. This is the first report that investigates adipogenic capacity, finding a potential role for IL-6 in mediating the process in IS vs IR tissue from obese non-white individuals. The clinical data confirmed previously reported heterogeneity of associated pathology, even among individuals susceptible to insulin resistance and with morbid obesity. SC pre-adipocytes of the IS subset preserved their capacity to differentiate, perhaps facilitating excess energy to be largely stored as triacylglycerols in this depot and lowering ectopic fat deposition, as indicated by the more favourable liver-function variables. Further, IS-derived pre-adipocytes exhibited a higher pro-adipogenic and lower anti-adipogenic gene expression profile. These differences between insulin sensitivity and insulin resistance, albeit subtle, may be a consequence of a preponderance of large adipocytes, prone to forming inflammatory foci, in the IR group [33], accounting for small but significant differences in adipogenic capacity in this cohort. Markers of oxidative stress were also elevated in the IR state. Thus the related scenario of inflammation and oxidative stress is a likely mediator of increased IL-6 secretion in this depot.

The positive correlation between IL-6 levels and SVF cell numbers suggests a mitogenic effect on all SVF cells (endothelial, epithelial, macrophages, pre-adipocytes) known to express its cell surface receptor or able respond to its soluble form (sIL-6R). IL-6 was selected as a prime candidate based on previous studies suggesting that chronic exposure of mice to IL-6 induces insulin resistance in skeletal muscle cells and hepatocytes [34, 35]. This study provides data that supports previous findings of elevated IL-6 expression in human fat cells from IR individuals [23, 36], and the reported positive correlation between secreted IL-6 and adipocyte resistance to insulin [37]. A negative correlation between secreted IL-6 and SC adipogenic capacity was apparent, perhaps consequent to IL-6 repression of *CEBP*α expression, as reported previously in hepatoma cells [38]. Additionally, *GATA-3*, shown previously to bind *CEBPα* as a critical step in inhibition of pre-adipocyte differentiation [39], was positively associated with secreted IL-6, suggesting that the ratio of the two nuclear factors in response to local secretion of IL-6 might be critical for modulating SC adipogenesis.

While IL-6 levels were inversely related to adipogenic capacity and treatment with IL-6 in vitro reduced adipogenic capacity, this reduction was equal in IS and IR cells (Fig. 4b, d). IR individuals may be expected to be ‘resistant’ to this cytokine because of possible prolonged exposure to it in vivo; however, this was not apparent in vitro, perhaps due to IL-6 receptor binding mode and subsequent signal transduction. IL-6 signals via a heterodimeric receptor complex consisting of ligand-binding IL-6 α-subunit (IL-6R) and a signal transducing subunit (gp130). In addition a ligand binding the soluble form of IL-6R (sIL-6R) is present in both blood and urine, and is an agonist. While both the membrane-bound IL-6R and sIL-6R bind the cytokine with equal affinities, the response to the IL-6/sIL-6R complex in vivo can be substantially different from the response to IL-6 alone [40]. sIL-6R is generated mainly by proteolytic cleavage of the extracellular domain of IL-6R by the metalloprotease ADAM 17 [41]. Interestingly, adipocyte differentiation is increased when ADAM 17 is knocked down [42]. SC adipose tissue from the human mammary depot demonstrated expression of IL-6R and gp130 protein in mature adipocytes but not in undifferentiated adipocyte precursor cells [43]. Blocking IL-6 trans-signalling in rodents prevents high-fat diet-induced adipose tissue macrophage (ATM) recruitment. While in mice this did not improve insulin resistance, in humans, in the same study, there were significant correlations between insulin resistance (as measured by the HOMA-IR), sIL-6R plasma concentrations and ATM accumulation [44]. We have assessed the expression of IL-6R and gp130 in differentiated adipocytes and found them to be comparable in insulin sensitivity and insulin resistance (data not shown). Thus, in vivo trans-signalling may largely explain the IR cohort’s inability to undergo adipogenesis. This is currently being investigated further in cells from IS and IR individuals.

Previous work has focused on studying purified pre-adipocytes following depletion of inflammatory cells [14, 20]. This study investigated the behaviour of pre-adipocytes within their natural niche where obesity-induced inflammation plays an integral role in their pathophysiology [20]. Therefore, depleting endothelial cells and/or leucocytes would potentially deprive them of their paracrine effects [45, 46]. Phenotypic characterisation of cellular expression of SVF-derived pre-adipocytes suggested a heterogeneity in the plasticity of SVF cells between insulin sensitivity and insulin resistance, perhaps accounting for the reduced adipogenic capacity seen in the IR individuals. Previous studies have shown impaired adipogenesis in obesity [14], as well as the presence of two pre-adipocyte subtypes with respect to their differentiation potential within human pre-adipocyte populations [47]. It was suggested that the presence of both subtypes allows plasticity of the progenitor pool over time in response to various stimuli, such as inflammatory cytokines, with long-term consequences for the cellular composition, function and adipogenic potential of fat depots [47].

Inherent features of pre-adipocytes were shown previously to persist in expanded in vitro cultures, including different size, lipoprotein binding, fatty acid transfer, protein secretion and response to insulin and lipolytic agents [36]. The observed ex vivo inherent impairment of SC adipogenic capacity in all IR individuals may be another feature that persists in vitro, and can explain the in vivo elevated levels of plasma triacylglycerols and the ectopic deposition in the liver seen in the IR group of this cohort and reported by others [48].

This study has several limitations. One important difference between the non-diabetic group (IS + IR) and type 2 diabetes mellitus group is their insulin sensitising and anti-inflammatory medication (metformin). Despite treatment, these individuals still show impairment of adipogenesis and perhaps if untreated, the difference in impairment of adipogenic capacity would be even greater. It is worth noting that none of the recruited participants with type 2 diabetes mellitus were being treated with thiazolidinediones (TZDs), thus a window for improvement with TZDs through enhancement of pre-adipocyte adipogenesis may still exist. Another possible limitation is the relatively small size of each of the three groups, although similar studies had enough power to detect differences in adipogenic capacity with increased BMI [14] and with insulin resistance in lean individuals [21].

We show that impaired SC adipogenesis resulting in a reduced capacity to store triacylglycerols and accommodate energy in a physiological manner marks obesity-associated IR and is mediated, at least in part, through local secretion of IL-6. These findings suggest that measures aimed at lowering tissue inflammation are likely to stave off insulin resistance. Understanding the mechanisms underlying greater adipogenesis in the IS group compared with the IR group, could validate the therapeutic potential of novel targets, such as *CEBPα/GATA3* ratio, as well as heterogeneity in cellular IL-6 signalling.

## Abbreviations

ALT: Alanine transaminase
AST: Aspartate aminotransferase
ATM: Adipose tissue macrophage
DBP: Diastolic blood pressure
FPG: Fasting plasma glucose
IL-6R: Ligand-binding IL-6 α-subunit
IR: Insulin-resistant
IS: Insulin-sensitive
PRX2: Peroxiredoxin 2
ROS: Reactive oxygen species
SC: Subcutaneous
sIL-6R: Soluble form of IL-6R
SOD1: Superoxide dismutase 1
SOD2: Superoxide dismutase 2
SVF: Stromal vascular fraction
TRX1: Thioredoxin
TZD: Thiazolidinedione

## References

[1] Bogardus C, Lillioja S, Mott DM, Hollenbeck C, Reaven G (1985). Relationship between degree of obesity and in vivo insulin action in man. Am J Physiol.

[2] Samocha-Bonet D, Chisholm DJ, Tonks K, Campbell LV, Greenfield JR (2012). Insulin-sensitive obesity in humans - a ʻfavorable fatʼ phenotype?. Trends Endocrinol Metab: TEM.

[3] Karelis AD, Faraj M, Bastard JP (2005). The metabolically healthy but obese individual presents a favorable inflammation profile. J Clin Endocrinol Metab.

[4] Stefan N, Kantartzis K, Machann J (2008). Identification and characterization of metabolically benign obesity in humans. Arch Intern Med.

[5] Bjorntorp P (1974). Effects of age, sex, and clinical conditions on adipose tissue cellularity in man. Metab Clin Exp.

[6] Spalding KL, Arner E, Westermark PO (2008). Dynamics of fat cell turnover in humans. Nature.

[7] Okuno A, Tamemoto H, Tobe K (1998). Troglitazone increases the number of small adipocytes without the change of white adipose tissue mass in obese Zucker rats. J Clin Invest.

[8] Tontonoz P, Hu E, Spiegelman BM (1994). Stimulation of adipogenesis in fibroblasts by PPARγ2, a lipid-activated transcription factor. Cell.

[9] Guilherme A, Virbasius JV, Puri V, Czech MP (2008). Adipocyte dysfunctions linking obesity to insulin resistance and type 2 diabetes. Nat Rev Mol Cell Biol.

[10] Radcke S, Dillon JF, Murray AL (2015). A systematic review of the prevalence of mildly abnormal liver function tests and associated health outcomes. Eur J Gastroenterol Hepatol.

[11] Gronthos S, Franklin DM, Leddy HA, Robey PG, Storms RW, Gimble JM (2001). Surface protein characterization of human adipose tissue-derived stromal cells. J Cell Physiol.

[12] Tchkonia T, Morbeck DE, Von Zglinicki T (2010). Fat tissue, aging, and cellular senescence. Aging Cell.

[13] Rosen ED, MacDougald OA (2006). Adipocyte differentiation from the inside out. Nat Rev Mol Cell Biol.

[14] Isakson P, Hammarstedt A, Gustafson B, Smith U (2009). Impaired preadipocyte differentiation in human abdominal obesity: role of Wnt, tumor necrosis factor-alpha, and inflammation. Diabetes.

[15] Salans LB, Knittle JL, Hirsch J (1968). The role of adipose cell size and adipose tissue insulin sensitivity in the carbohydrate intolerance of human obesity. J Clin Invest.

[16] Arner E, Westermark PO, Spalding KL (2010). Adipocyte turnover: relevance to human adipose tissue morphology. Diabetes.

[17] McLaughlin T, Lamendola C, Coghlan N (2014). Subcutaneous adipose cell size and distribution: relationship to insulin resistance and body fat. Obesity.

[18] Ryden M, Andersson DP, Bergstrom IB, Arner P (2014). Adipose tissue and metabolic alterations: regional differences in fat cell size and number matter, but differently: a cross-sectional study. J Clin Endocrinol Metab.

[19] Furukawa S, Fujita T, Shimabukuro M (2004). Increased oxidative stress in obesity and its impact on metabolic syndrome. J Clin Invest.

[20] Gustafson B, Gogg S, Hedjazifar S, Jenndahl L, Hammarstedt A, Smith U (2009). Inflammation and impaired adipogenesis in hypertrophic obesity in man. Am J Physiol Endocrinol Metab.

[21] Acosta JR, Douagi I, Andersson DP (2016). Increased fat cell size: a major phenotype of subcutaneous white adipose tissue in non-obese individuals with type 2 diabetes. Diabetologia.

[22] Flower L, Gray R, Pinkney J, Mohamed-Ali V (2003). Stimulation of interleukin-6 release by interleukin-1beta from isolated human adipocytes. Cytokine.

[23] Rotter V, Nagaev I, Smith U (2003). Interleukin-6 (IL-6) induces insulin resistance in 3T3-L1 adipocytes and is, like IL-8 and tumor necrosis factor-alpha, overexpressed in human fat cells from insulin-resistant subjects. J Biol Chem.

[24] Mohamed-Ali V, Goodrick S, Rawesh A (1997). Subcutaneous adipose tissue releases interleukin-6, but not tumor necrosis factor-alpha, in vivo. J Clin Endocrinol Metab.

[25] Madani R, Karastergiou K, Ogston NC (2009). RANTES release by human adipose tissue in vivo and evidence for depot-specific differences. Am J Physiol Endocrinol Metab.

[26] Lagathu C, Bastard JP, Auclair M, Maachi M, Capeau J, Caron M (2003). Chronic interleukin-6 (IL-6) treatment increased IL-6 secretion and induced insulin resistance in adipocyte: prevention by rosiglitazone. Biochem Biophys Res Commun.

[27] Grundy SM, Brewer HB, Cleeman JI (2004). Definition of metabolic syndrome: report of the National Heart, Lung, and Blood Institute/American Heart Association conference on scientific issues related to definition. Circulation.

[28] Bunnell BA, Estes BT, Guilak F, Gimble JM (2008). Differentiation of adipose stem cells. Methods Mol Biol.

[29] Kafienah W, Mistry S, Williams C, Hollander AP (2006). Nucleostemin is a marker of proliferating stromal stem cells in adult human bone marrow. Stem Cells.

[30] Lee MJ, Wu Y, Fried SK (2012). A modified protocol to maximize differentiation of human preadipocytes and improve metabolic phenotypes. Obesity.

[31] Gustafson B, Hammarstedt A, Hedjazifar S, Smith U (2013). Restricted adipogenesis in hypertrophic obesity: the role of WISP2, WNT, and BMP4. Diabetes.

[32] Mohamed-Ali V, Flower L, Sethi J (2001). β-Adrenergic regulation of IL-6 release from adipose tissue: in vivo and in vitro studies. J Clin Endocrinol Metab.

[33] Cinti S, Mitchell G, Barbatelli G (2005). Adipocyte death defines macrophage localization and function in adipose tissue of obese mice and humans. J Lipid Res.

[34] Nieto-Vazquez I, Fernandez-Veledo S, de Alvaro C, Lorenzo M (2008). Dual role of interleukin-6 in regulating insulin sensitivity in murine skeletal muscle. Diabetes.

[35] Klover PJ, Zimmers TA, Koniaris LG, Mooney RA (2003). Chronic exposure to interleukin-6 causes hepatic insulin resistance in mice. Diabetes.

[36] Tchkonia T, Thomou T, Zhu Y (2013). Mechanisms and metabolic implications of regional differences among fat depots. Cell Metab.

[37] Bastard JP, Maachi M, Van Nhieu JT (2002). Adipose tissue IL-6 content correlates with resistance to insulin activation of glucose uptake both in vivo and in vitro. J Clin Endocrinol Metab.

[38] Foka P, Irvine SA, Kockar F, Ramji DP (2003). Interleukin-6 represses the transcription of the CCAAT/enhancer binding protein-alpha gene in hepatoma cells by inhibiting its ability to autoactivate the proximal promoter region. Nucleic Acids Res.

[39] Tong Q, Tsai J, Tan G, Dalgin G, Hotamisligil GS (2005). Interaction between GATA and the C/EBP family of transcription factors is critical in GATA-mediated suppression of adipocyte differentiation. Mol Cell Biol.

[40] Peters M, Muller AM, Rose-John S (1998). Interleukin-6 and soluble interleukin-6 receptor: direct stimulation of gp130 and hematopoiesis. Blood.

[41] Chalaris A, Rabe B, Paliga K (2007). Apoptosis is a natural stimulus of IL6R shedding and contributes to the proinflammatory trans-signaling function of neutrophils. Blood.

[42] Wang Y, Kim KA, Kim JH, Sul HS (2006). Pref-1, a preadipocyte secreted factor that inhibits adipogenesis. J Nutr.

[43] Path G, Bornstein SR, Gurniak M, Chrousos GP, Scherbaum WA, Hauner H (2001). Human breast adipocytes express interleukin-6 (IL-6) and its receptor system: increased IL-6 production by β-adrenergic activation and effects of IL-6 on adipocyte function. J Clin Endocrinol Metab.

[44] Kraakman MJ, Kammoun HL, Allen TL (2015). Blocking IL-6 trans-signaling prevents high-fat diet-induced adipose tissue macrophage recruitment but does not improve insulin resistance. Cell Metab.

[45] Villaret A, Galitzky J, Decaunes P (2010). Adipose tissue endothelial cells from obese human subjects: differences among depots in angiogenic, metabolic, and inflammatory gene expression and cellular senescence. Diabetes.

[46] Karastergiou K, Mohamed-Ali V (2010). The autocrine and paracrine roles of adipokines. Mol Cell Endocrinol.

[47] Tchkonia T, Tchoukalova YD, Giorgadze N (2005). Abundance of two human preadipocyte subtypes with distinct capacities for replication, adipogenesis, and apoptosis varies among fat depots. Am J Physiol Endocrinol Metab.

[48] Murguia-Romero M, Jimenez-Flores JR, Sigrist-Flores SC (2013). Plasma triglyceride/HDL-cholesterol ratio, insulin resistance, and cardiometabolic risk in young adults. J Lipid Res.

